# Fibulin-1 Is Increased in Asthma – A Novel Mediator of Airway Remodeling?

**DOI:** 10.1371/journal.pone.0013360

**Published:** 2010-10-13

**Authors:** Justine Y. Lau, Brian G. Oliver, Melissa Baraket, Emma L. Beckett, Nicole G. Hansbro, Lyn M. Moir, Steve D. Wilton, Carolyn Williams, Paul S. Foster, Philip M. Hansbro, Judith L. Black, Janette K. Burgess

**Affiliations:** 1 Cooperative Research Centre for Asthma and Airways, Sydney, New South Wales, Australia; 2 Discipline of Pharmacology, The University of Sydney, Sydney, New South Wales, Australia; 3 Woolcock Institute of Medical Research, Sydney, New South Wales, Australia; 4 Centre for Asthma and Respiratory Disease and Hunter Medical Research Institute, The University of Newcastle, Newcastle, New South Wales, Australia; 5 Centre for Neuromuscular and Neurological Disorders, University of Western Australia, Perth, Western Australia, Australia; 6 Lung Institute of Western Australia and the Centre for Asthma, Allergy and Respiratory Research, University of Western Australia, Perth, Western Australia, Australia; The University of Hong Kong, Hong Kong

## Abstract

**Background:**

The extracellular matrix is a dynamic and complex network of macromolecules responsible for maintaining and influencing cellular functions of the airway. The role of fibronectin, an extracellular matrix protein, is well documented in asthma. However, the expression and function of fibulin-1, a secreted glycoprotein which interacts with fibronectin, has not been reported. Fibulin-1 is widely expressed in basement membranes in many organs including the lung. There are four isoforms in humans (A–D) of which fibulin-1C and 1D predominate. The objective of this study was to study the expression of fibulin-1 in volunteers with and without asthma, and to examine its function *in vitro*.

**Methodology/Principal Findings:**

We used immunohistochemistry and dot-blots to examine fibulin-1 levels in bronchial biopsies, bronchoalveolar lavage fluid and serum. Real-time PCR for fibulin-1C and 1D, and ELISA and western blotting for fibulin-1 were used to study the levels in airway smooth muscle cells. The function of fibulin-1C was determined by assessing its role, using an antisense oligonucleotide, in cell proliferation, migration and wound healing. A murine model of airway hyperresponsiveness (AHR) was used to explore the biological significance of fibulin-1. Levels of fibulin-1 were significantly increased in the serum and bronchoalveolar lavage fluid of 21 asthmatics compared with 11 healthy volunteers. In addition fibulin-1 was increased in asthma derived airway smooth muscle cells and fibulin-1C contributed to the enhanced proliferation and wound repair in these cells. These features were reversed when fibulin-1C was suppressed using an antisense oligomer. In a mouse model of AHR, treatment with an AO inhibited the development of AHR to methacholine.

**Conclusions:**

Our data collectively suggest fibulin-1C may be worthy of further investigation as a target for airway remodeling in asthma.

## Introduction

Asthma is a chronic inflammatory disorder of the airways characterized by variable and reversible airflow obstruction and airway hyperresponsiveness (AHR). A key feature of asthmatic airways is remodeling which involves thickening of the airway wall, altered deposition of extracellular matrix (ECM) proteins [1], [2] and increased airway smooth muscle (ASM) mass. These structural changes may result from an aberrant repair process in the lung, which includes increased proliferation of the ASM cells [3], [4]. Whilst current treatments control the symptoms of asthma, they are unable to fully prevent or reverse airway remodeling.

The ECM maintains airway function and structure by providing mechanical support in addition to constituting a dynamic and complex network that influences cellular function [5]. The ECM deposited by asthma derived ASM cells is altered such that increased amounts of collagen I and laminin [6], [7], [8], as well as fibronectin (FN) are produced which mediate a range of cellular interactions including migration, growth and differentiation.

Levels of the profibrotic cytokine transforming growth factor β (TGFβ) are elevated in the bronchoalveolar lavage (BAL) fluid in asthma [9], and are increased in bronchial tissue [10]. TGFβ stimulated FN deposition is also enhanced in asthma derived bronchial epithelium and fibroblasts [11], [12].

Fibulin-1 (FBLN-1), a secreted glycoprotein, assists in stabilizing the ECM. It associates with FN and a variety of other ECM proteins including laminin and fibrinogen [13]. Mice deficient in FN and FBLN-1 die perinatally due to abnormal lung development [14]. FBLN-1 expression has been reported in human lung tissue using microarray technology, however, FBLN-1 was not verified by PCR or at the protein level, nor were functional studies carried out [15], [16], [17]. In addition, Laprise *et al.*, reported reduced FBLN-1D expression in asthma derived bronchial biopsies compared with those derived from non-asthmatics [15]. However, the function of FBLN-1 in the lungs and its role in asthma is unknown.

Four isoforms of FBLN-1 have been identified to date in humans, designated FBLN-1A, 1B, 1C, and 1D [18]. These isoforms are splice variants which possess different C-terminal sequences. Although a global increase of FBLN-1 is associated with tumor cells [19], the FBLN-1 isoforms may have distinct, important roles in regulating cellular behavior. Importantly, the isoform FBLN-1C was not reported in previous studies that examined human lung tissue. Recently, Hergeth *et al.*, showed that the proliferation of blood CD34+ progenitor cells is inhibited by FBLN-1C [20]. We, and others, have shown that the proliferation of ASM cells derived from asthmatic individuals is enhanced compared with those from non-asthmatic individuals [4], [21], [22]. Given the contribution of the ASM cell to remodeling in asthma, we hypothesized that FBLN-1C may play an important regulatory role in the lung.

The aim of this study was to assess the production of FBLN-1C by asthmatic and non-asthmatic volunteers, and further, to determine its function in isolated cell systems, and in an *in vivo* model of airway hyperresponsiveness.

## Materials and Methods

### Ethics Statement

The study was approved by the Ethics Review Committee of the South West Sydney Area Health Service, Royal Prince Alfred Hospital and The University of Sydney human research ethics committee. All volunteers provided written informed consent.

### Study population

Samples obtained from a total of 64 asthmatics and 63 non-asthmatics were studied. Non-asthmatic ASM cells were obtained from resected lung following thoracotomy or transplantation. Bronchial biopsies, BAL fluid, serum and ASM cells were obtained from volunteers with intermittent, mild persistent or moderate persistent atopic asthma, according to GINA guidelines [23], or healthy volunteers. Some samples were collected prior to and after 7 weeks of treatment with an inhaled corticosteroid (fluticasone propionate (200 or 1000 mcg daily)). Healthy volunteers had no history of asthma or other lung disease and normal spirometry. Medication and smoking history was not available for all patients in this study. The details of all the individuals from whom samples were obtained are shown in Table S1.

### Collection and preparation of samples

#### Bronchoalveolar lavage fluid and serum

BAL fluid was obtained by means of flexible fibreoptic bronchoscopy from 20 asthmatics and 11 non-asthmatics. Specimens were collected via normal saline lavage of the segmental airways and alveolar spaces (BAL) and endobronchial biopsy of the mucosal lining of the airway. To remove mucus and cells, the BAL fluid was filtered through sterile gauze and centrifuged at 580 g for 5 min. The acellular supernatant was stored at −80°C until used.

In addition, venous blood was collected from the forearm of volunteers, 44 asthmatics and 15 non-asthmatics and was stored in aliquots at −20°C until used.

#### Airway smooth muscle

ASM cells were isolated from 31 asthmatics and 44 non-asthmatics. ASM cells were isolated as previously described [3]. Briefly, bronchial airways were dissected from the surrounding parenchyma and blood vessels and cut longitudinally. Subsequently, the airways were washed in ethanol and sterile Hank's balanced salt solution before dissection under a dissecting microscope. Bronchial mucosa biopsies were placed in Hank's balanced salt solution (Invitrogen, Carlsbad, CA, USA) for immediate dissection of the ASM bundles for culture. The bronchial epithelial cell layer was removed with fine forceps exposing the visible smooth muscle bundles which were then dissected free from the surrounding tissue. The collected smooth muscle bundles were, placed in Hank's balanced salt solution and centrifuged at 150×g for 10 min. Isolated pieces of muscle were placed into 25 cm^2^ vented tissue culture flasks (BD Biosciences, North Ryde, Australia) containing 2.5 mLs Dulbecco's modified eagle's medium (DMEM) (Invitrogen) supplemented with 10% fetal bovine serum (FBS) (JRH biosciences, Brooklyn, Australia) and 2 U/mL penicillin, 2 µg/mL streptomycin, and 250 ng/mL amphotericin B (2% antibiotics) (Invitrogen) and placed in a humidified CO_2_ incubator (5% CO_2_ in air) and maintained at 37°C. Weekly, the medium was aspirated and replaced with DMEM supplemented with 5% FBS, 1% GlutaMAX™-I supplement (Invitrogen) and 1% antibiotics (Growth medium).

### Airway smooth muscle cell culture

ASM cells were seeded at a density of 1×10^4^ cells/cm^2^ with growth medium for 9 d at 37°C/5% CO_2_. On day 5, the medium was replenished with fresh growth medium. Cells were quiesced in 0.1% bovine serum albumin (BSA) (Sigma Aldrich, St Louis, MO, USA), 1% GlutaMAX™-I supplement, 1% antibiotics in DMEM (quiescing medium) for 3 d and treated with either quiescing medium or 10 ng/mL TGFβ (R&D Systems, Minneapolis, MN, USA) in quiescing medium for 8 (mRNA analysis) or 24 (protein analysis) h.

### Dot Blot

The levels of soluble FBLN-1 in serum, BAL fluid and ASM cell supernatants were measured using dot blot techniques. The protein levels of FBLN-1C were not measured as no isoform specific antibody was available at the time of experimentation. As a strong, specific signal for FBLN-1 was detectable in FBS this was used as a positive control for these experiments. ASM cells were cultured as described above in 96 well plates. After 24 h of treatment, supernatants were removed and stored at −20°C until analyzed.

Polyvinylidene fluoride (PVDF) membrane (Immobilon–P, 0.45 µm pore size) (Millipore, Billerica, MA, USA) was cut and pre-wet with 100% methanol for 10 min and then titrated into phosphate buffered saline (PBS) for 15 min before being placed in the blot apparatus. Human serum (50 µL, diluted 1∶1000 in PBS), BAL fluid (no dilution), supernatant (no dilution), FBS (diluted 1∶2 in PBS) (positive control) or PBS (negative control) were loaded into separate slots. A vacuum was applied until the samples were evacuated. The membrane was washed 3 times with PBS and the flow through removed with the aid of the vacuum.

The membrane was blocked in 5% (w/v) BSA/PBS-Tw (blocking buffer) for 1 h at room temperature. Primary antibody, monoclonal mouse anti-human FBLN-1 antibody (1 µg/mL in 1% (w/v) BSA in PBS-Tw) (Alexis Biochemicals, San Diego, CA, USA), was added and incubated for 1 h at room temperature. The membrane was then washed 3 times with PBS-Tw and a biotinylated goat anti-mouse secondary antibody (1∶20 000 dilution in 1% (w/v) BSA/PBS-Tw) (Millipore) added, and incubated for 1 h at room temperature. After washing 3 times with PBS-Tw, streptavidin horseradish peroxidise (HRP) (0.5 µg/mL in 1% (w/v) BSA/PBS-Tw) (R&D Systems) was added for 1 h at room temperature. The membrane was washed with PBS-Tw for 15 min and chemiluminescence (West Dura Extended duration substrate kit, Pierce, Rockford, IL, USA) was visualized. The image was captured and densitometric data were obtained using Kodak MI software (version 4.0.5) (Kodak Scientific Imaging Systems, Rochester, NY, USA). We normalized the values for FBLN-1 by first subtracting the background and then expressing it as a fold change from the positive control, 50% (v/v) FBS.

### Fibulin-1C downregulation by transcript specific targeted antisense oligmer

ASM cells were seeded at a density of 1×10^4^ cells/cm^2^ in growth medium for 1 d at 37°C/5% CO_2_. A FBLN-1C antisense oligomer (AO) consisting of 2′-0-methyl modified bases on a phosphorothioate backbone (5′ CUUGCUAAGACUUUAUUAACGCC) was used to downregulate FBLN-1C mRNA and protein, and a scrambled AO (5′ AUUUUGUCUGAAACCCUGUAAAGAG) was used as the negative control. We recognized the high homology between the different FLBN-1 isoforms and therefore we targeted the transcript specific final exon of FBLN-1C. The AO directed against FBLN-1C targeted the 1C polyadenylation site in the 3′UTR and was specific for that transcript only. If aligned with the FBLN-1D polyadenylation site, the FBLN-1C 23mer contains 15 mis-matches to the 1D sequence, precluding any potential cross annealing. We have previously shown that a highly effective AO loses all activity with 3 consecutive mismatches [24].

For 6 well plates (1.6 mLs of medium), 200 nM of either the scrambled AO or FBLN-1C AO, 19.012 µg of enhancer buffer and 3.34 µg of Effectene® transfection reagent was added to transfect the cells according to the manufacturer's instructions for 72 h (Effectene® transfection reagent, Qiagen, Doncaster, Australia). For different sized plates, the concentration of the transfection reagent was adjusted. Cells were quiesced in quiescing medium for a further 3 d.

To produce ECM with suppressed FBLN-1C expression, ASM cells were seeded in 75 cm^2^ flasks and transfected as described above. ASM cells were reseeded into 24 or 96 well plates maintaining the presence or absence of transfection reagent for a further 3 d. ASM cells were lyzed using 0.016 mM sterile NH_4_OH at 37°C for 20 min as previously described [25], [26]. The wells were washed five times with sterile PBS and stored in sterile PBS at −20°C.

### Real Time PCR

ASM cells were seeded in 6 well plates as described above. After 8 h of treatment with or without 10 ng/mL TGFβ, total cellular RNA was extracted using the NucleoSpin RNA II kit according to the manufacturer's instructions (Macherey-Nagel GmbH & Co, Duren, Germany). RNA was eluted in 50 µL of RNase free water compared with 60 µL as specified by the manufacturer.

Reverse transcription of total RNA and real time PCR was performed as described previously [27]. Predeveloped assay on demand primers were used as follows FBLN-1: Hs00242545_m1; FBLN-1C: Hs00242546_m1; FBLN-1D Hs00197774_m1 (Applied Biosystems, Foster City, CA, USA). The data were expressed as a fold change relative to the non-stimulated (0.1% BSA) sample.

### Western blotting

ASM cells were seeded in 6 well plates as described above. After 24 h of treatment with or without 10 ng/mL TGFβ, cells were lyzed using 50 µL of protein lysis buffer (0.9% (w/v) NaCl, 20 mM Tris HCl (pH 7.6), 0.1% (v/v) Triton X-100, 1mM phenylmethanesulphonylfluoride (PMSF) and 1% (v/v) protease inhibitor cocktail (Calbiochem, Gibbstown, NJ, USA)) and stored at −20°C until required.

Western blotting was carried out as described previously [28] with the following modifications. We mixed and denatured 30 µL of protein with 6 µL of loading buffer (2% sodium dodecyl sulphate (SDS), 7.5% glycerol, 31.25 mM Tris-HCl (pH 6.8), 0.0025% bromophenol blue, 200 mM DTT) at 95°C for 5 min. As a positive control, 25 µg of SK-BR-3, a human breast carcinoma cell line known to express FBLN-1 (Abgent, San Diego, CA, USA) was used. The PVDF membrane was blocked with blocking buffer (5% (w/v) BSA in 0.05% (v/v) Tween-20 in PBS) followed by the addition of antibodies diluted in 1% BSA/PBS-Tw. Antibodies used to analyze FBLN-1 levels were a monoclonal mouse anti-human FBLN-1 antibody (1 µg/mL) followed by a biotinylated secondary goat anti-mouse antibody (1 in 20 000 dilution) and streptavidin HRP (0.5 µg/mL) each incubated for 1 h at room temperature. To analyze glyceraldehyde 3-phosphate dehydrogenase (GAPDH), a housekeeping monoclonal mouse anti-human GAPDH antibody (0.002 ng/mL) (Santa Cruz Biotechnology) was incubated for 2 h followed by a goat anti-mouse secondary antibody (0.4 µg/mL) (Santa Cruz Biotechnology) for another h.

The values for FBLN-1 were normalized against those obtained for GAPDH on the same membrane and are expressed as a fold change relative to non-stimulated cells (0.1% BSA). To enable the comparison of the basal level of FBLN-1, the levels of FBLN-1 were normalized against the positive control, whole cell lysates of the SK-BR-3 human breast carcinoma cell line.

### ECM Enzyme Linked Immunosorbent Assay

ASM cells were seeded in 96 well plates and grown in 100 µL of growth medium as described above and treated with or without 10 ng/mL TGFβ for 24 h. ASM cells were lyzed using 0.016 mM NH_4_OH at 37°C for 20 minutes. The wells were washed five times with PBS and stored with 50 µL/well of PBS at −20°C until analyzed.

The deposition of FBLN-1 in the ECM was assessed using an ECM ELISA according to methods described previously [29]. On the day of the ELISA, after defrosting and another wash, a seven point standard curve, ranging from 0.3125% to 20% (v/v) FBS in PBS, was loaded on to each plate. All antibodies were added at 50 µL/well and diluted in 1% BSA/PBS-Tw. Monoclonal mouse anti-human FBLN-1 antibody (2 µg/mL) was used as the primary antibody, and the isotype control was a mouse IgM (2 µg/mL). For measurement of FBLN-1 or its isotype control, a biotinylated sheep anti-mouse antibody (5 µg/mL) (Millipore) was then added followed by steptavidin HRP (0.5 µg/mL). ECM was quantified by reading the plate at 405 nm using a Wallac-Victor 2 plate reader and software (Perkin Elmer Life Sciences, Rowville, Australia).

The data are expressed as fold change relative to non-stimulated cells (0.1% BSA). In addition, the deposition of FBLN-1 in the ECM was compared with standard curves. Samples and standard curves were analyzed using Microplate Manager (Version 4.0, Bio-Rad Laboratories, Hurcules, CA). FBLN-1 is expressed as a percentage of the FBS standard.

### Detection of fibulin-1 and fibronectin in airway smooth muscle cells

ASM cells were seeded in eight well glass chamber slides (Nalge Nunc, Rochester, NY, USA) and cultured as described above. After 24 h of treatment, the cells were fixed using cold 4% paraformaldehyde for 20 min at 4°C.

The slides were blocked in 1% (w/v) BSA in PBS for 30 min at room temperature and washed in PBS three times. A polyclonal rabbit anti-human FBLN-1 antibody (4 µg/mL in PBS) (Santa Cruz Biotechnology, Santa Cruz, CA, USA) and a polyclonal rabbit anti-human FN antibody (3 µg/mL in PBS) (Sigma Aldrich, St Louis, MO, USA) were conjugated with Alexa Fluor 555 (12 µg/mL in PBS) and Alexa Fluor 647 (1 µg/mL in PBS), respectively, in 10 µL PBS using a Zenon Rabbit IgG Labeling Kit (Molecular Probes, Heidelberg, Australia). After 5 min incubation in the dark, a non-specific rabbit IgG (Alexa Fluor 555: 300 µg/mL and Alexa Fluor 647: 25 µg/mL, both in PBS) was added to each sample and incubated in the dark for a further 5 min. The two antibody samples were combined in PBS (antibody∶zenon complex). One hundred µL of the antibody∶zenon complex was added to each well of the chamber slide and incubated for 1.5 h at room temperature in the dark. After incubation, slides were washed in PBS three times and refixed in cold 4% paraformaldehyde for 20 min at 4°C. Slides were washed in PBS three times prior to being coverslipped using 15% (v/v) glycerol in PBS, pH 8.0. The cells were visualized using filters 575–615 and 650 nm for Alexa 555 and 647, respectively, using a Zeiss EC Plan-Neofluar 40×0.75 NA dry objective on a laser scanning confocal microscope (Zeiss LSM 510 Meta Axioplan 2).

### Wound healing

#### Airway smooth muscle cells

ASM cells were seeded in 24 well plates in the presence or absence of the FBLN-1C AO. A scrambled AO and Effectene® alone were used as negative controls. A wound, approximately 1 cm in length was made in the cell monolayer with a yellow pipette tip (P200, QSP, Pathtech, Preston, Australia). The wells were washed twice with sterile PBS and replenished with either quiescing medium or 10 ng/mL TGFβ in quiescing medium for up to 72 h.

#### Airway smooth muscle cells on a non-autologous extracellular matrix

ECM in which FBLN-1C was suppressed was produced using the FBLN-1C AO and prepared as described above. ASM cells were then reseeded on top of this non-autologous matrix as previously described [6]. A wound was made in the cell monolayer as described for ASM cells.

#### Wound healing assay

The wound was observed using phase contrast microscopy with an Olympus microscope (model CK2) and images of the entire wound were taken at 0, 16, 24, 48 and 72 h post wound induction with an Olympus digital camera (model C-4000).

Images of each wound were merged into a single image, such that the image contained the whole wound using Adobe Photoshop CS3 (Adobe Systems). The area of each wound at each different time point was measured in pixels, using ImageJ 1.37v software. The percentage of wound closure compared with the initial wound size was calculated using the equation.
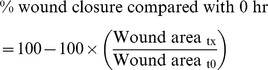
Where _tx_ is the wound area at a given time point and _t0_ is the initial wound area

### Cell proliferation

Cell proliferation was assessed by MTT [3-(4,5-dimethylthiazol-2-yl)-2,5-diphenyltetrazolium] (Sigma Aldrich, St Louis, MO, USA) assay as previously described [30]. FBLN-1C expression in the ECM was suppressed by AO treatment and ASM cells were reseeded on the ECM as previously described [6]. ASM cell proliferation was measured after 72 h, with the addition of 0.5 mg/mL of MTT 5 h before the experimental end point. Absorbance was measured at test wavelength 570 nm and reference wavelength 655 nm.

### Cell migration

ASM cell migration was examined using a modified Boyden chamber protocol. To prepare ECM ASM cells were cultured and transfected with and without FBLN-1C AO, in cell culture inserts (uncoated, 8.0 µm pore size, transparent polyethylene terephthalate membrane, BD Biosciences). After 4 days of growth the cell culture inserts were washed once and then frozen at −20°C in sterile PBS to lyze the cells and expose the ECM. The inserts were stored at −20°C prior to use. ASM cells were seeded in 75 cm^2^ flasks at a density of 1×10^4^ cells/cm^2^ in growth medium at 37°C/5% CO_2_. After 4 d of growth, ASM cells were quiesced in quiescing medium for 3 d. Twenty h prior to the assay, cells were treated with either quiescing medium or 10 ng/mL TGFβ in quiescing medium.

Cell culture inserts with a pre-laid ECM generated from ASM cells were defrosted and washed an additional four times in sterile PBS to remove cell debris, and once in DMEM. ASM cells from the 75 cm^2^ flasks were trypsinized, centrifuged, and washed in 5 mL sterile PBS twice. After the final centrifugation, the cells were resuspended in quiescing medium maintaining the absence or presence of 10 ng/mL TGFβ. The cells were seeded into the inner chamber of the cell culture inserts as per the manufacturer's instructions (BD Biosciences). Growth medium was used as a chemoattractant. In addition to the specified controls, a no cell control for each matrix type was also used which consisted of quiescing medium added to the exposed ECM in the inner chamber and growth medium to the outer chamber.

ASM cells were allowed to migrate for 4 h at 37°C/5% CO_2_ then labeled and dissociated with a cell dissociation/Calcein-AM solution (1× cell dissociation solution (Bio Scientific Pty Ltd, Gymea, Australia), 2.4 µg Calcein AM (Molecular Probes) diluted in sterile water) according to the manufacturer's instructions. The cells were labeled for a total of 1.25 h before quantification using fluorescence (485 nm excitation and 520 nm emission on the SpectraMax M2 plate reader (Molecular Devices, Camberwell, Australia).

The fluorescence value of the ‘no cell control’ was subtracted from corresponding samples of the same matrix type. Data are expressed as fold change relative to ‘no chemoattractant’ sample.

### Generation, treatment and assessment of a mouse model of airway hyperresponsiveness

Adult BALB/c mice (6 to 8 weeks old) were administrated 200 ng of recombinant human TGFβ (R&D Systems, Minneapolis, MN, USA) intratracheally on days 0 and 1 to induce AHR. Briefly, prior to anesthesia, the mice were warmed using a heat lamp. Mice were anesthetized by IV injection with 12.5 mg/kg Alfaxalone (Jurox, NSW, Australia) before being suspended vertically and orotracheally intubated with a 22-gauge flexible plastic catheter (Terumo Sureflo, Hospital Supplies of Australia). Endotracheal positioning was confirmed by palpation of the tracheal rings with the catheter tip. Mice were administered 40 µL of PBS with or without 200 ng TGFβ. Animals remained vertical for 1 to 2 min after administration to ensure the inoculums remained in the lungs.

A series of AOs targeting FBLN-1 (5′ CACUUGGCGCACGACACCUGGGGA; 5′ UGAACUUGAUCCACUCACCCUCAGG; and 5′ UUUUCUCUUGGCGGAAGCUGCAGA) were used in equal parts to silence the expression of FBLN-1 in the mice, and a scrambled AO (5′ AUUUUGUCUGAAACCCUGUAAAGAA) was used as a negative control. The mice were anesthetized with isofluorane (4%) and treated with 40 µL of PBS alone, or containing 40 µg of FBLN-1 specific or scrambled AO intranasally. Treatment occurred daily for the first 7 days, and then 3 times weekly for the following three weeks.

AHR was assessed, *in vivo*, by measuring changes in transpulmonary resistance and dynamic compliance using a Buxco (Buxco, Willmington, NC, USA) invasive mouse plethysmograph on day 29 [31]. Mice were anesthetized with an intraperitoneal injection of ketamine/xylazine (80–100 mg/kg and 10 mg/kg respectively) and cannulated via the trachea with an 18 gauge metal cannula. Mice were then mechanically ventilated (150 strokes/min, 175 µL stroke volume). Volume changes due to thoracic expansion with ventilation were measured by a transducer connected to the plethysmograph flow chamber. Once stabilized, the mice were challenged with 10 µL of saline, followed by increasing concentrations of methacholine (0.625, 1.25, 2.5, 5, 10 and 20 mg/mL), aerosolized by an ultrasonic nebuliser and administered directly to the lungs via the inspiratory line. Each aerosol was delivered for a period of 5 min, during which pressure and flow data were continuously recorded. A BioSystemXA (Buxco Electronics, Willmington, NC, USA) was used to calculate pulmonary resistance and compliance. Peak values were taken as the maximum response to the concentration of methacholine being tested. Data are expressed as the percentage change over the PBS control.

### Statistics

For seeding experiments, in which the characteristics of the ASM cells were being investigated, all data collected from ASM cells derived from a single volunteer seeded on different ECMs were averaged. Likewise, where the characteristics of the ECM were investigated, all results obtained when assorted ASM cells were seeded on matrices deposited by ASM derived from a single volunteer were averaged.

Gaussian distribution was determined by the Kolmogorov-Sminov test, where an alpha level greater than 0.05 was considered the threshold. For experiments that compared cells or ECM derived from different volunteers, analysis was conducted using an unpaired t-test. For experiments comparing more than two factors within each disease group, analysis was conducted using one-way ANOVA (Bonferroni post test). For experiments comparing two or more factors and between disease groups, analysis was performed using two-way ANOVA (Bonferroni post test). A *p* value of less than 0.05 (p≤0.05) was considered statistically significant.

Data were analyzed using GraphPad Prism version 5.00 (GraphPad Software, San Diego, CA, USA).

## Results

### Fibulin-1 levels are increased in serum and BAL fluid from asthmatic volunteers

**Figure 1:**
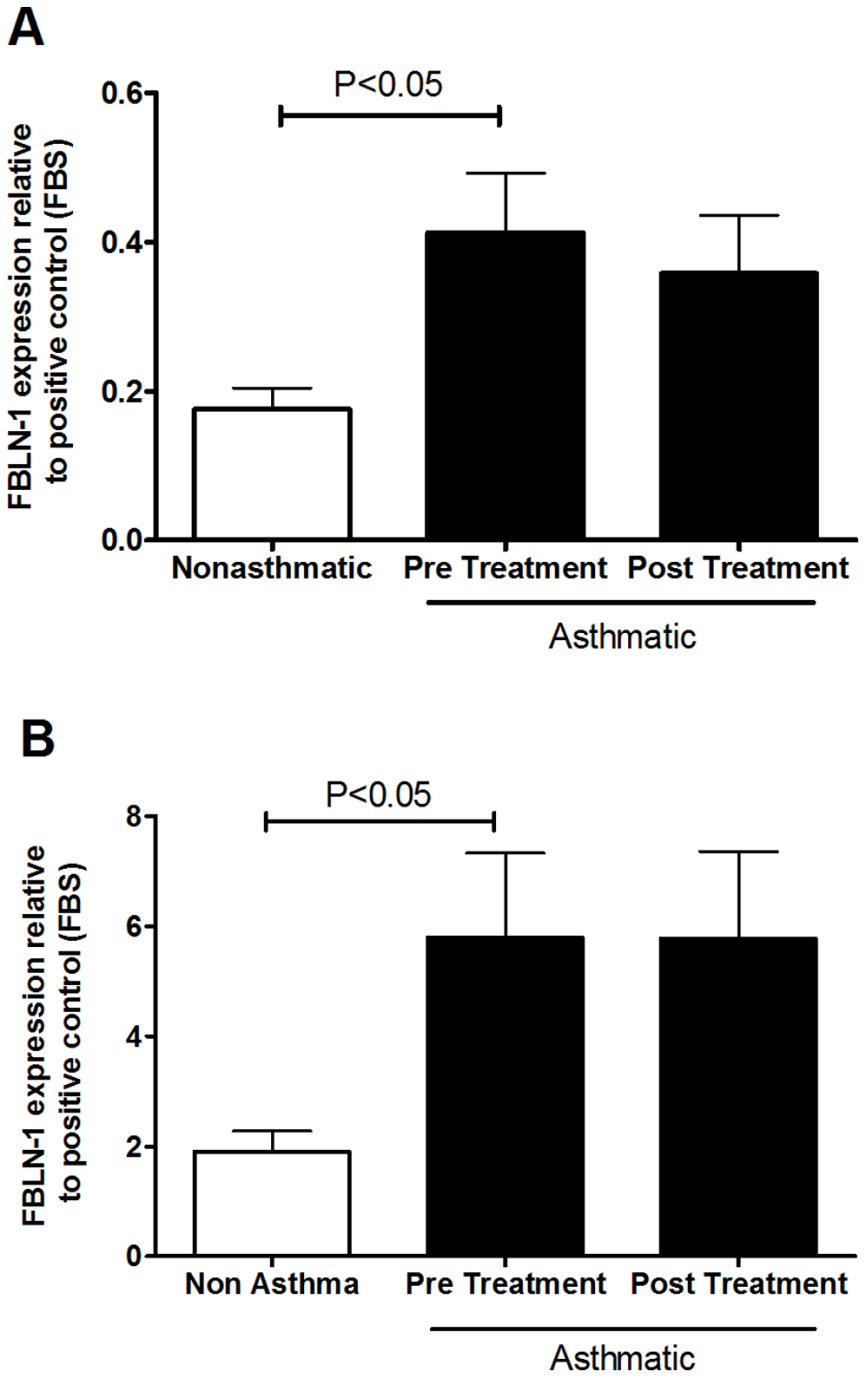
FBLN-1 is increased in the serum and BAL of asthmatics. FBLN-1 levels in serum (A) or BAL fluid (B) from non-asthmatic (white) (n = 11) or asthmatic (black, pre and post corticosteroid treatment, n = 20) volunteers. Densitometric values are expressed relative to the positive control FBS. Data are expressed as mean ± SEM.

We measured FBLN-1 levels in serum (figure 1A) and BAL fluid (figure 1B) from 20–44 asthmatic and 11–15 non-asthmatic volunteers by dot blot analysis. FBLN-1 levels were greater in volunteers with asthma compared with those without asthma and these levels were not altered by *in vivo* corticosteroid treatment (figure 1A & B). There was no correlation between the FBLN-1 level detected in the serum or BAL from the asthmatic volunteers and their FEV_1_/FVC ratio (data not shown).

### TGFβ upregulates fibulin-1C mRNA and FBLN-1 protein levels in ASM cells derived from people with asthma

**Figure 2:**
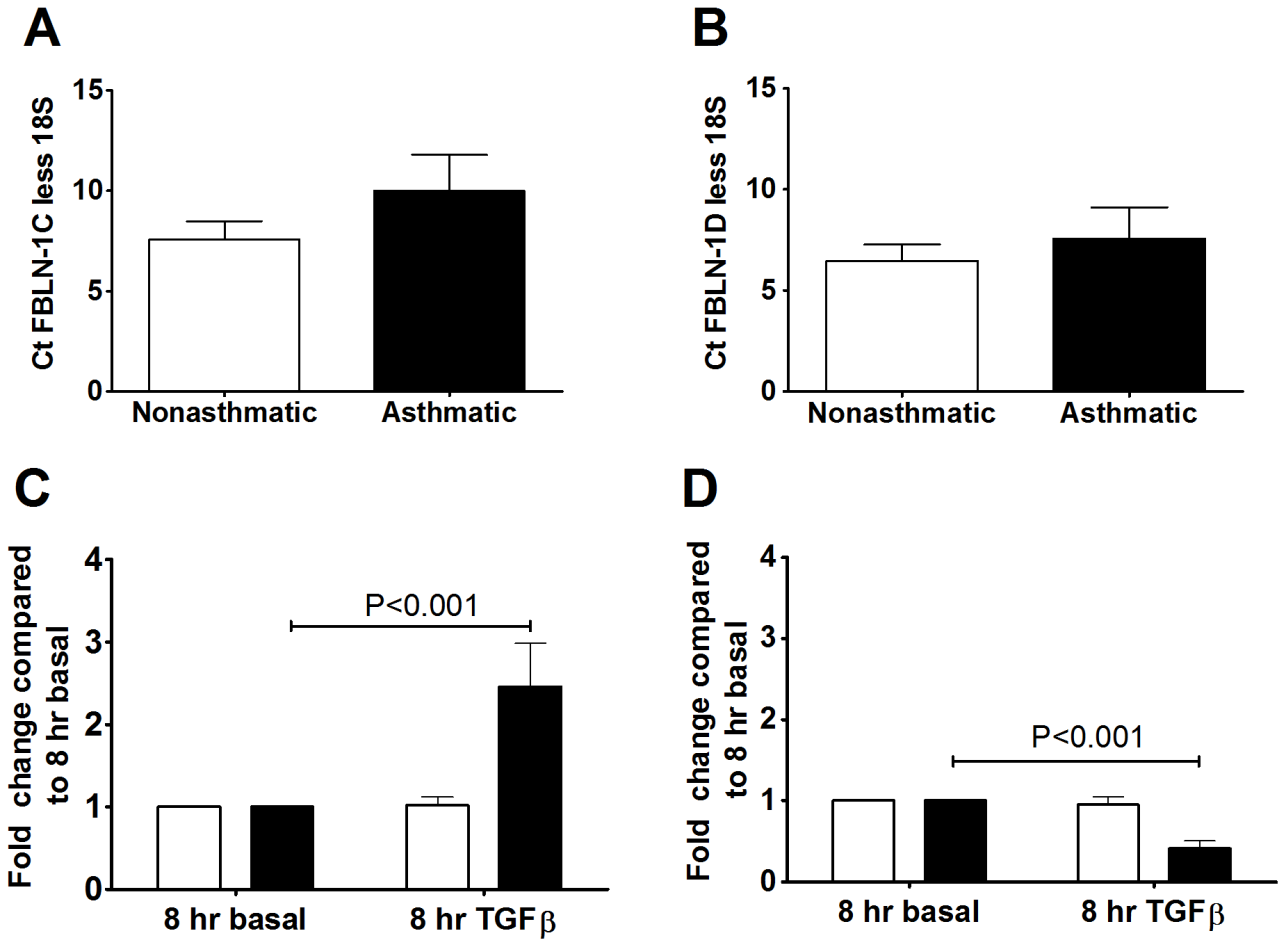
TGFβ induced FBLN-1C mRNA in asthmatic ASM cells. Basal expression of FBLN-1C (A) and FBLN-1D (B) mRNA (expressed as cycle threshold difference between FBLN-1C or D and 18s rRNA) from ASM cells collected from non-asthmatic (white bars n = 12) or asthmatic (black bars, n = 8) volunteers, or expression following treatment for 8 h with TGFβ of FBLN-1C (C) and FBLN-1D (D) (data were normalized to 18s rRNA and are expressed relative to BSA at 8 h). Data are expressed as mean ± SEM.

**Figure 3:**
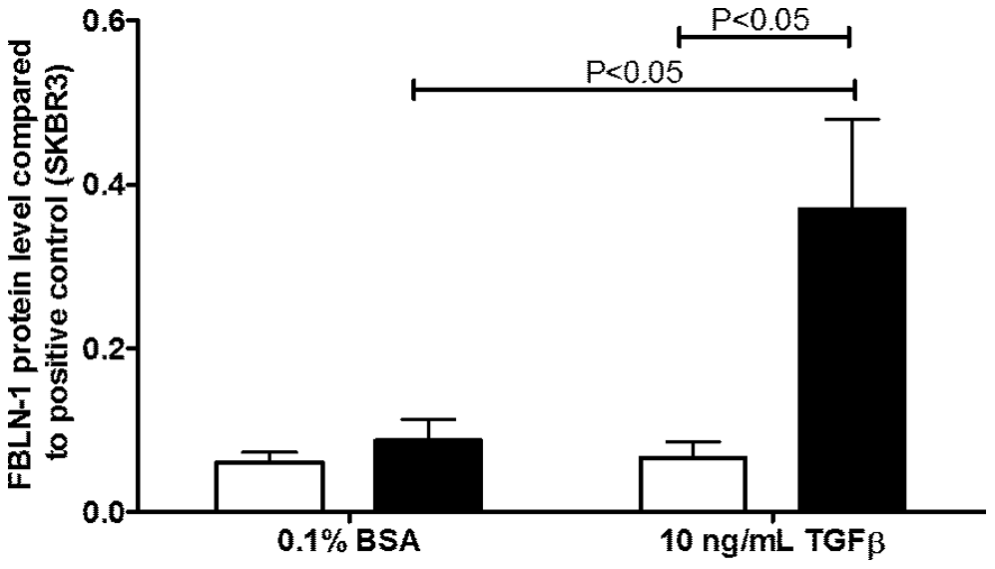
TGFβ induced FBLN-1 protein in asthmatic ASM cells. FBLN-1 protein was detected by western blot following TGFβ stimulation for 24 h of ASM cells collected from non-asthmatic (white bars, n = 4) or asthmatic (black bars, n = 4) volunteers. Data were normalized to the house keeping protein GAPDH and are expressed relative to a positive control of the cell line SKBR3. Data are expressed as mean ± SEM.

**Figure 4:**
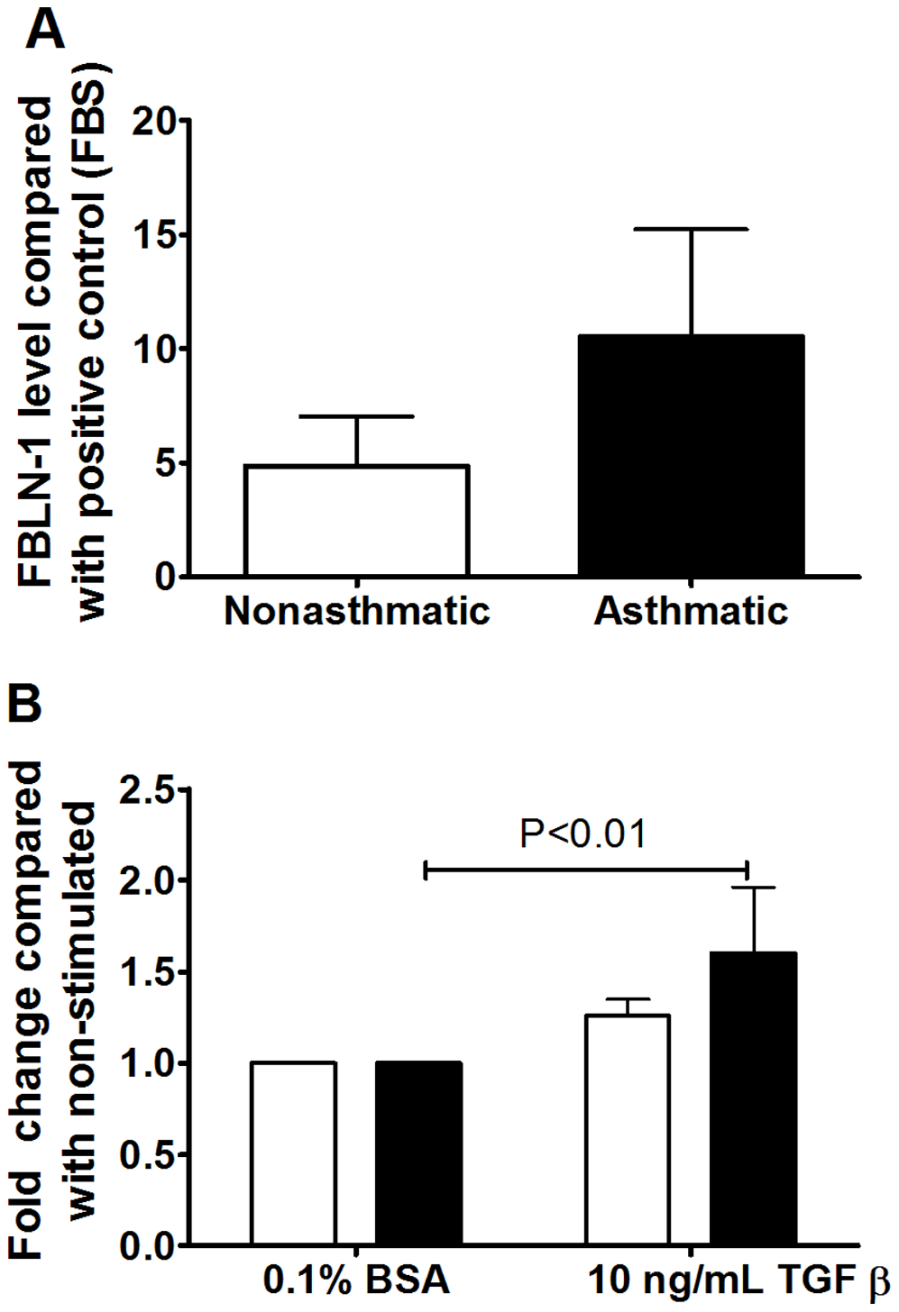
TGFβ induced FBLN-1 in ECM produced by asthmatic ASM cells. Basal expression of FBLN-1 (A) deposited by ASM cells collected from non-asthmatic (white bars, n = 10) or asthmatic (black bars, n = 10) volunteers, or FBLN-1 levels following treatment for 24 h with TGFβ (B). Data were normalized to a positive control (50% FBS) and are expressed relative to 0.1% BSA (non-stimulated). Data are expressed as mean ± SEM.

There were no differences in the basal expression of FBLN-1C or 1D mRNA between asthmatic and non-asthmatic ASM cells (figure 2A & B). TGFβ induced FBLN-1C mRNA in the ASM cells derived from asthmatic volunteers (P<0.001) but did not increase the level of FBLN-1D mRNA, rather it decreased it (p<0.001, figure 2 C & D). Neither FBLN-1C nor 1D expression was altered by TGFβ in the non-asthmatic cells. FBLN-1 protein was induced by TGFβ stimulation of ASM cells from asthmatic but not non-asthmatic volunteers as assessed by western blotting (figure 3). It was not possible to determine if this was due to an increase in FBLN-1C protein as a commercial antibody, which could distinguish between the FBLN-1 isoforms, was not available at the time of this study. We also analyzed ECM FBLN-1 deposition from ASM cells by ELISA. Levels of FBLN-1 in the ECM were the same in unstimulated non-asthmatic and asthmatic ECM (figure 4A). However, TGFβ increased FBLN-1 deposition in the ECM from asthmatic ASM cells (P<0.01, figure 4B). In addition to measuring FBLN-1 deposited in the ECM we also measured it in cell culture supernatants (soluble FBLN-1). ASM cell derived soluble FBLN-1 levels from the two patient groups were not different, either basally or after stimulation with TGFβ (data not shown).

### Rate of wound repair is increased in Asthmatic ASM cells

**Figure 5:**
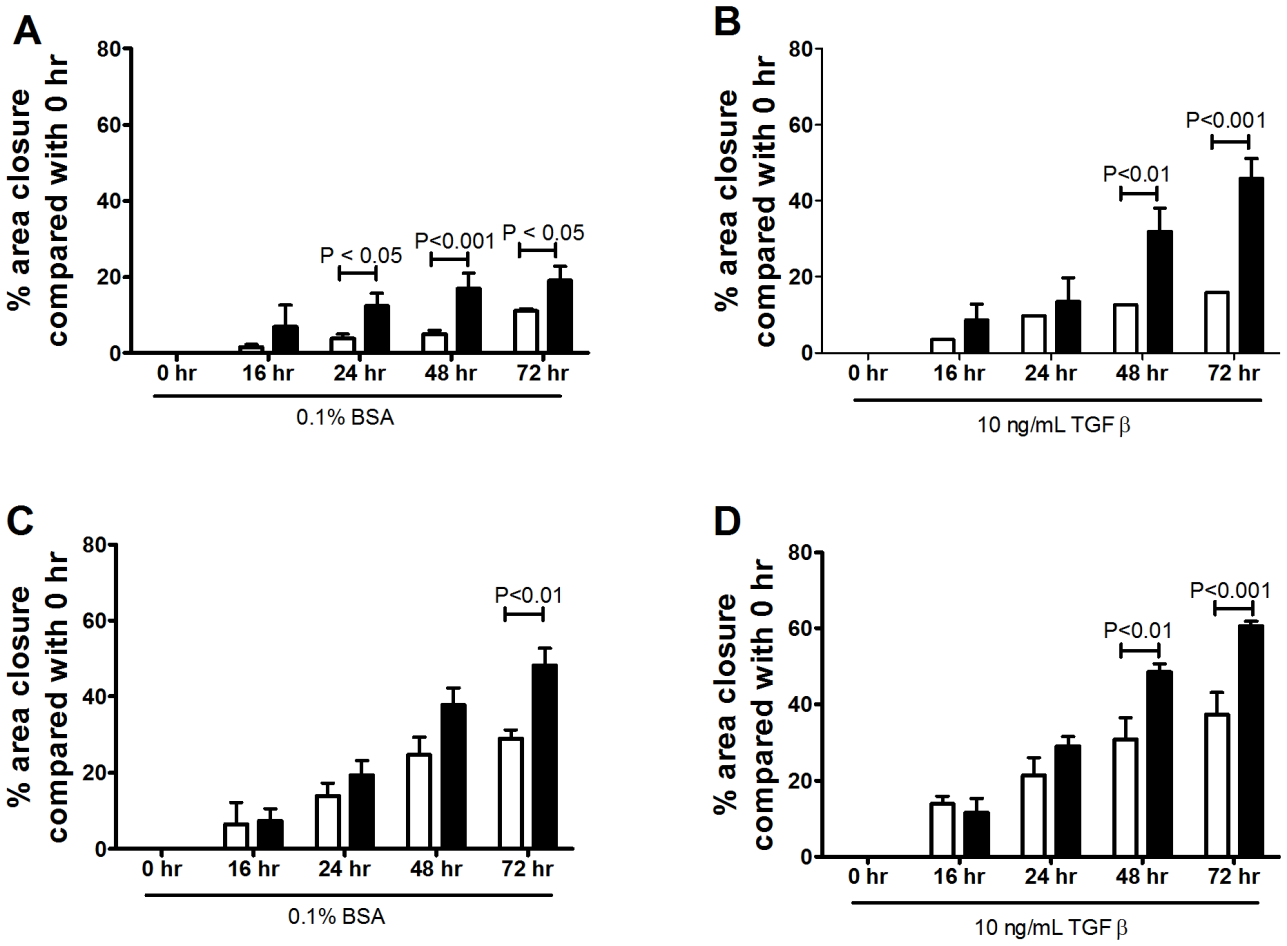
Wound healing is enhanced in asthmatic ASM cells. The degree of closure, measured over time, of a 1cm wound induced in non-asthmatic (white, n = 3–4) or asthmatic (black, n = 3–4) unstimulated (A) or TGFβ stimulated (B) ASM cells. The cells were reseeded on an ECM deposited by an asthmatic ASM cell line in the absence (C) or presence (D) of TGFβ. Data are expressed as mean ± SEM.

In a standard wound healing assay [32], 24 h post wound induction, the rate of wound closure was significantly greater in asthma derived ASM (12.4±3.4%) when compared with non-asthma derived ASM (4.2±1.2%, (P<0.05). The increased rate of wound closure by asthmatic ASM cells was maintained in the presence of BSA up to 72 h (figure 5A). TGFβ stimulation enhanced the ability of the ASM cells to close a wound made in the cell layer but this was still greater in the asthmatic ASM cells than the non-asthmatic cells (figure 5B). The enhanced capacity of the asthmatic cells was, in part, driven by the ECM deposited by the cells, as reseeding non-asthmatic cells on a matrix deposited by asthmatic cells increased the capacity of the non-asthmatic cells to close the wound (figure 5C & D).

### Functional role of fibulin-1

**Figure 6:**
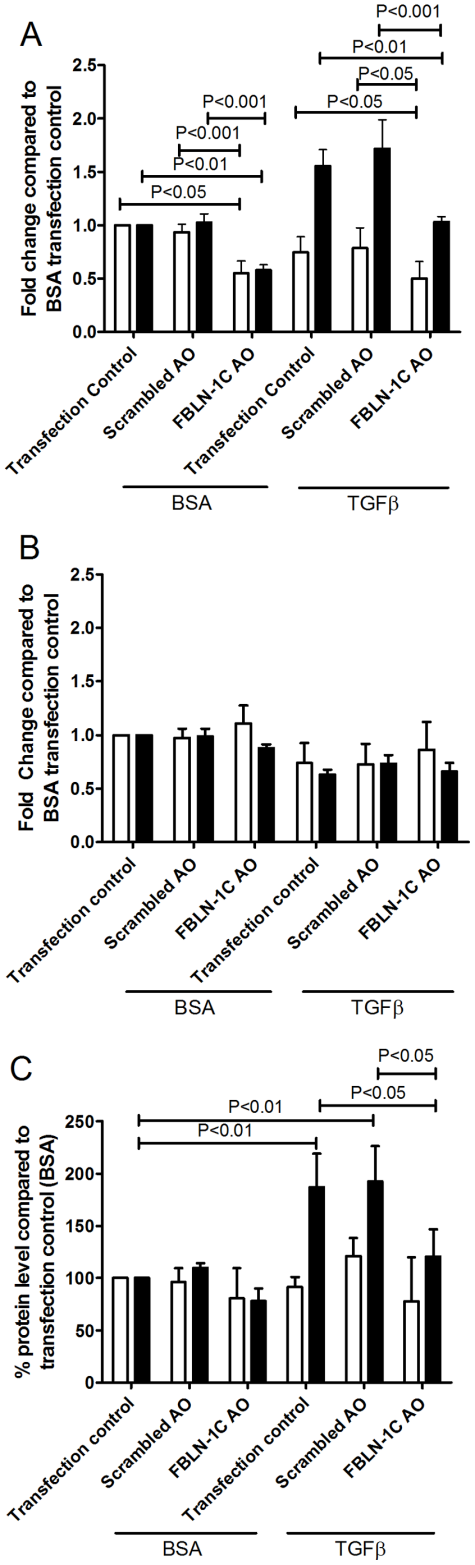
Efficiency and specificity of FBLN-1C AO. Inhibition of FBLN-1C (A) or FBLN-1D (B) mRNA following transfection with the FBLN-1C AO or a scrambled AO in ASM cells collected from non-asthmatic (white bars, n = 4) or asthmatic (black bars, n = 4) volunteers basally or following treatment for 8 h with TGFβ (data were normalized to 18s rRNA and are expressed relative to the transfection control at 8 h). FBLN-1 cellular and ECM deposited protein from ASM cells inhibited by a FBLN-1C specific AO (C). Data were normalized to a positive control (50% FBS) and are expressed relative to the transfection control. Data are expressed as mean ± SEM.

**Figure 7:**
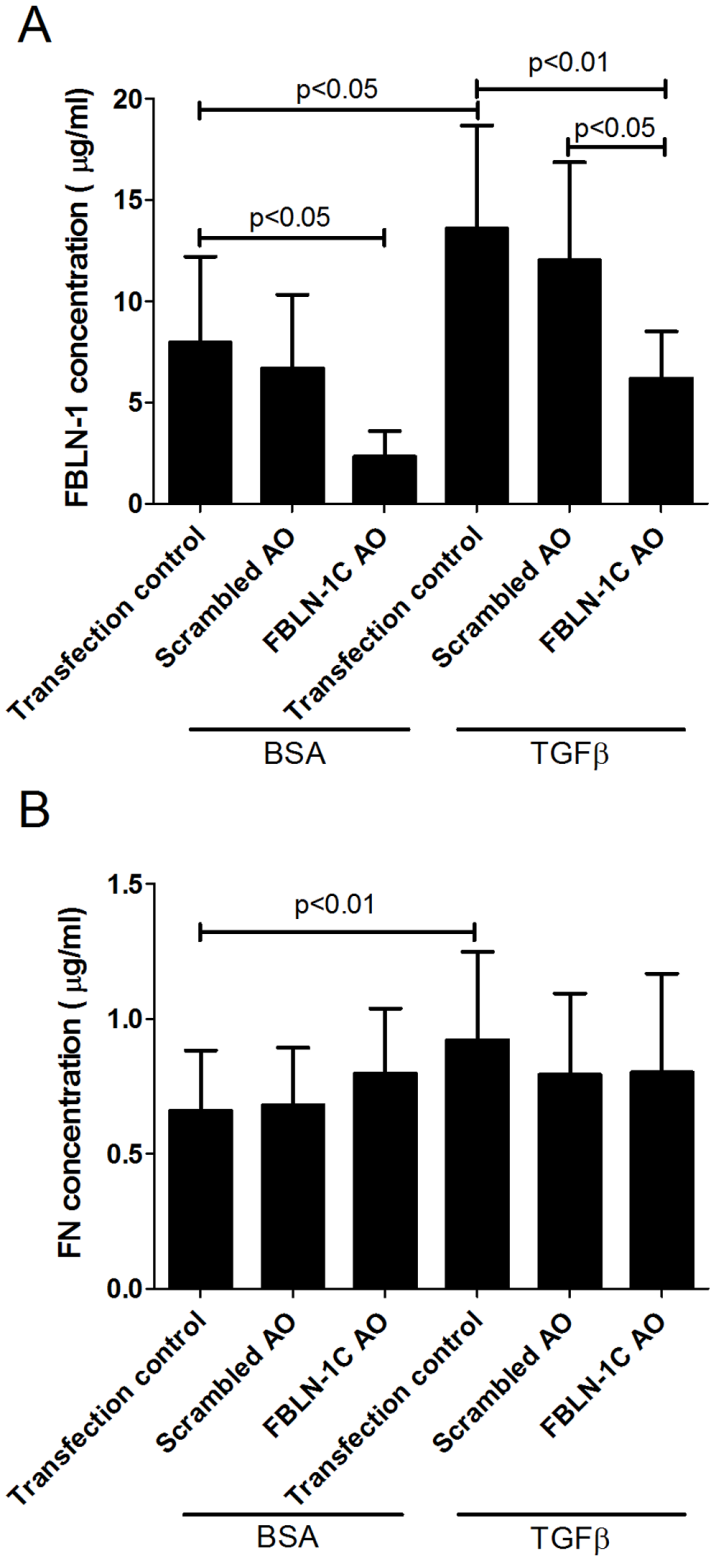
FBLN-1C AO does not alter FN deposition in ECM. Inhibition of ECM deposited FBLN-1 (A) or FN (B) following transfection with a FBLN-1C AO or a scrambled AO in ASM cells from asthmatic volunteers (n = 4) basally or following treatment with TGFβ for 24 h. Data are expressed as mean ± SEM. Concentrations were obtained by interpolation of standard curves.

To delineate the role of FBLN-1C in regulating the behavior of ASM cells we silenced its expression using a specific AO. Transfection of the FBLN-1C specific AO decreased FBLN-1C mRNA expression in asthmatic and non-asthmatic ASM derived cells compared with the transfection control (Effectene®) (P<0.05, P<0.01 respectively, figure 6A) whilst there were no changes in the mRNA levels of FBLN-1D (figure 6B). The scrambled AO had no effect on FBLN-1C or FBLN-1D mRNA expression. The two fold increase in FBLN-1 protein induced by TGFβ, detected using western blot, was maintained in the presence of the transfection control (Effectene®) and the scrambled AO. However the FBLN-1C AO almost completely abolished the TGFβ induced increase in protein (figure 6C). This did not occur when the ECM was derived from non-asthmatic cells. The increased FBLN-1 in the presence of TGFβ, detected in the ECM deposited by asthmatic ASM cells, was inhibited by the FBLN-1C specific AO (figure 7A).

**Figure 8:**
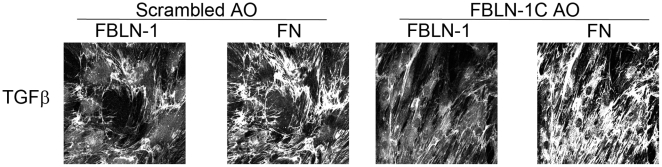
FBLN-1C AO inhibits FBLN-1 but not FN deposition by asthmatic ASM cells. ASM cells grown on glass coverslips in the presence of either a FBLN-1 specific AO or a scrambled AO were stimulated with TGFβ and FBLN-1 and FN were detected using antibodies labeled with different fluorochromes. Images are representative of results from 4 ASM cell lines.

Fibronectin mRNA (table 1) and protein levels (figures 7B & 8) were increased in the presence of TGFβ in the asthmatic ASM cells but were unchanged by the FBLN-1C AO. We did not examine the FN levels in non-asthmatic ASM cells as we had not seen an induction of FBLN-1 in these cells.

**Table 1:** mRNA expression of fibronectin in FBLN-1 AO transfected asthmatic ASM cells in the presence of TGFβ measured by quantitative real time PCR.

Transfection	Asthmatic ASM cells
Effectene®	1.28 (±0.08) [P<0.05]
Scrambled AO	1.50 (±0.17) [P<0.05]
FBLN-1C specific AO	1.57 (±0.21) [P<0.05]

Cells were derived from asthmatic (n = 6) volunteers and treated with Effectene® only, or transfected with 200 nM scrambled AO or FBLN-1C specific AO for 72 h. Cells were treated with either 0.1% BSA or 10 ng/mL TGFβ for 8 h. Expression levels were normalised to the housekeeping gene 18S. Data are expressed as fold change compared with 0.1% BSA values and shown as mean ± SEM [P value]. P values compared with their respective non-stimulated (0.1% BSA) values.

#### Fibulin-1 is involved in wound repair in asthma derived ASM cells

**Figure 9:**
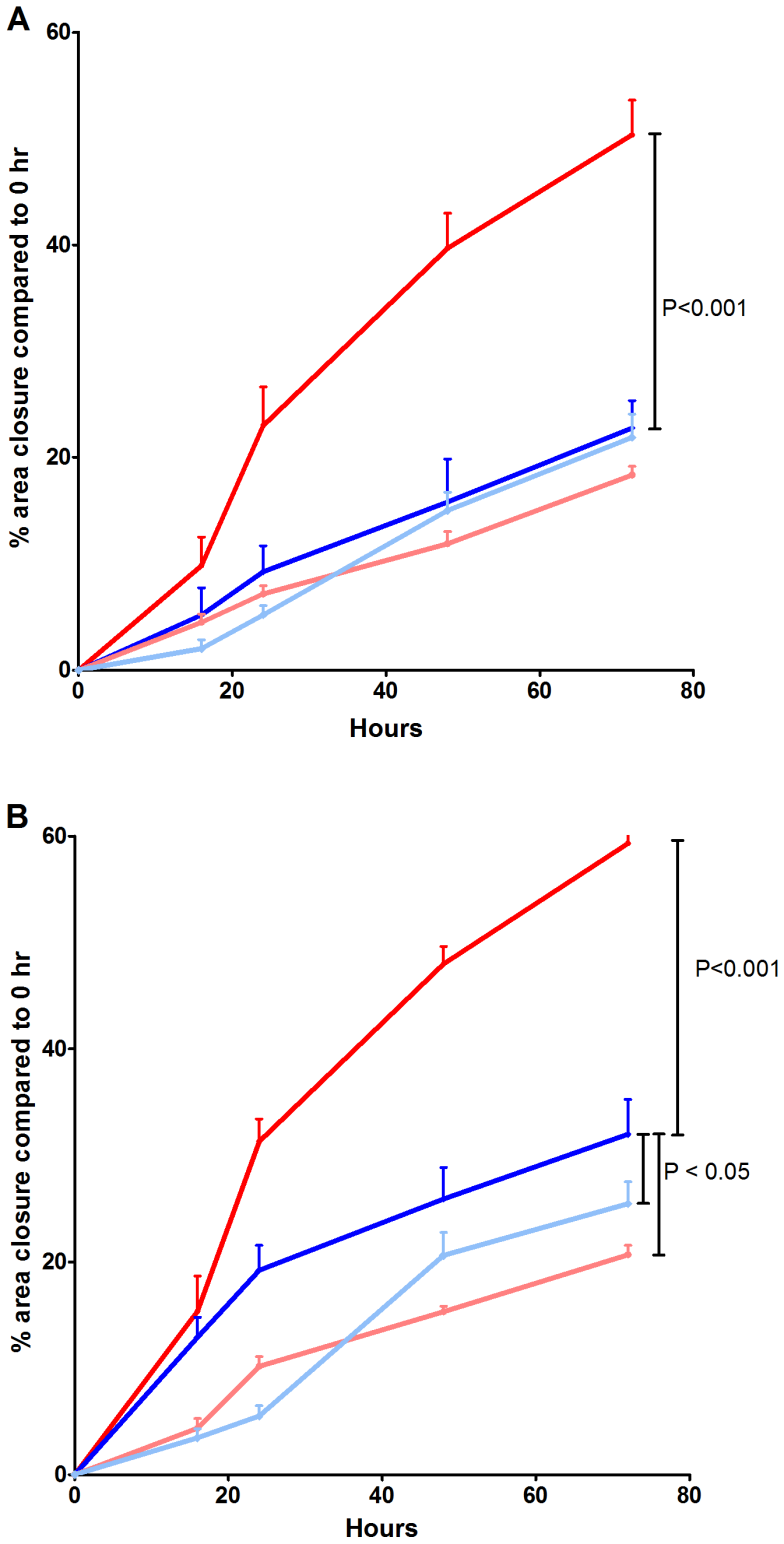
FBLN-1C promotes wound healing in asthmatic ASM cells. The degree of closure, measured over time, of a 1cm wound induced in non-asthmatic (n = 6–7) or asthmatic (n = 6–7) ASM cells reseeded on an ECM deposited by an asthmatic ASM cell line in the presence (dark red line (asthmatic) and dark blue line (non-asthmatic)) or absence (resulting from treatment with a FBLN-1C AO, light red line (asthmatic) and light blue line (non-asthmatic)) of FBLN-1C. Wound healing rates were measured basally (0.1% BSA) (A) or following stimulation with TGFβ (B). Data are expressed as mean ± SEM.

The FBLN-1C AO decreased the enhanced wound closure of asthmatic ASM cells both in the presence and absence of TGFβ (figure 9A & B). However this only occurred in the presence of TGFβ in non-asthma derived ASM cells (figure 9A & B). Transfection with a scrambled AO did not affect wound closure (data not shown).

#### Fibulin-1 is involved in cell proliferation in asthma derived ASM cells

**Figure 10:**
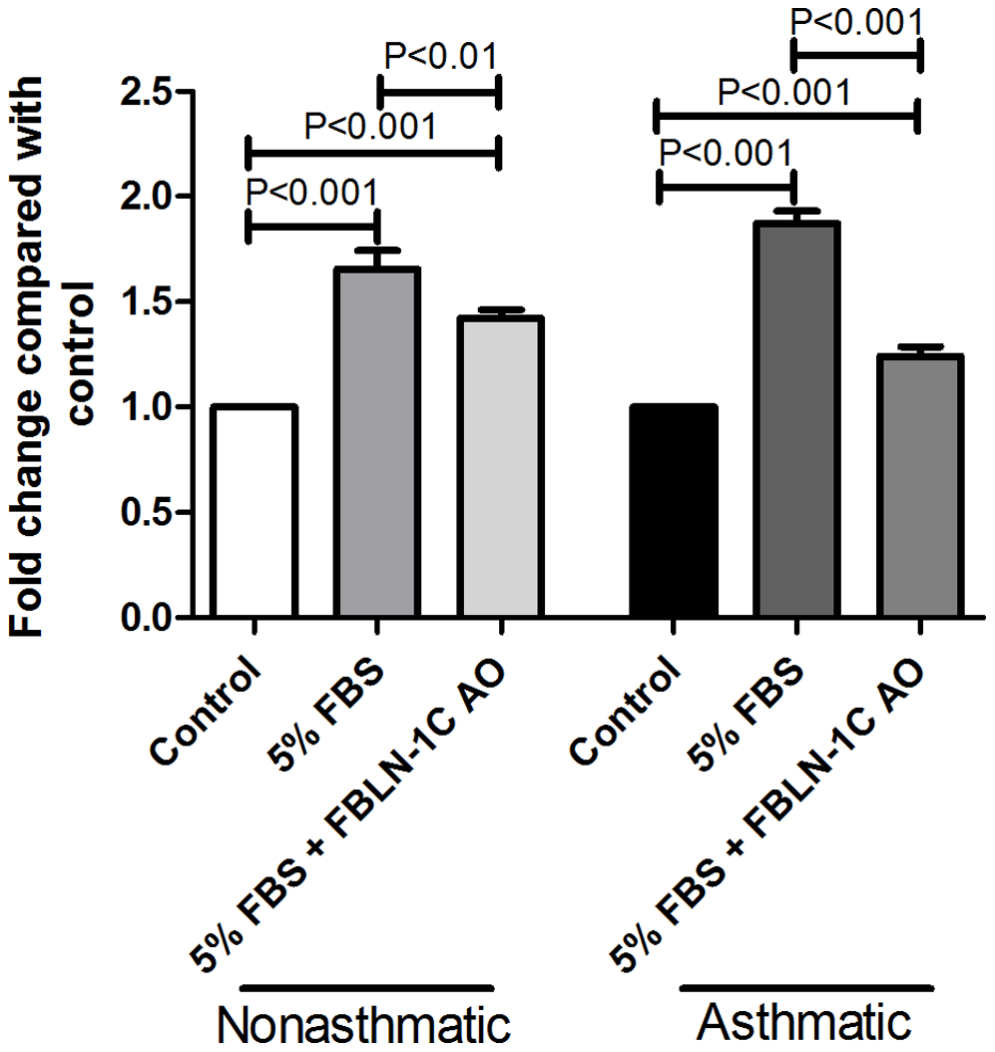
FBLN-1C promotes proliferation of ASM cells. FBLN-1C in the ECM deposited by an asthmatic ASM cell line was inhibited by a FBLN-1C specific AO. ASM cells from non-asthmatic (n = 4) or asthmatic (n = 4–5) volunteers were reseeded on the ECM and the effect on cell proliferation in response to 5% FBS was determined. Data are expressed as mean ± SEM.

Consistent with previous reports [3], [4], we confirmed that proliferation was increased in asthma derived ASM cells (data not shown) and also when asthmatic ASM cells (compared with non-asthmatic cells) were seeded on an asthma derived ECM [6]. Importantly, proliferation of asthmatic ASM cells was decreased when the cells were seeded onto an ECM produced by FBLN-1C AO treated cells (figure 10). Proliferation of non-asthmatic ASM cells was also suppressed when the cells were reseeded on a matrix produced by asthmatic ASM cells transfected with the FBLN-1C AO (figure 10). Transfection with the scrambled AO did not alter ASM cell proliferation (data not shown).

#### Fibulin-1 is not involved in migration of asthma derived ASM

**Figure 11:**
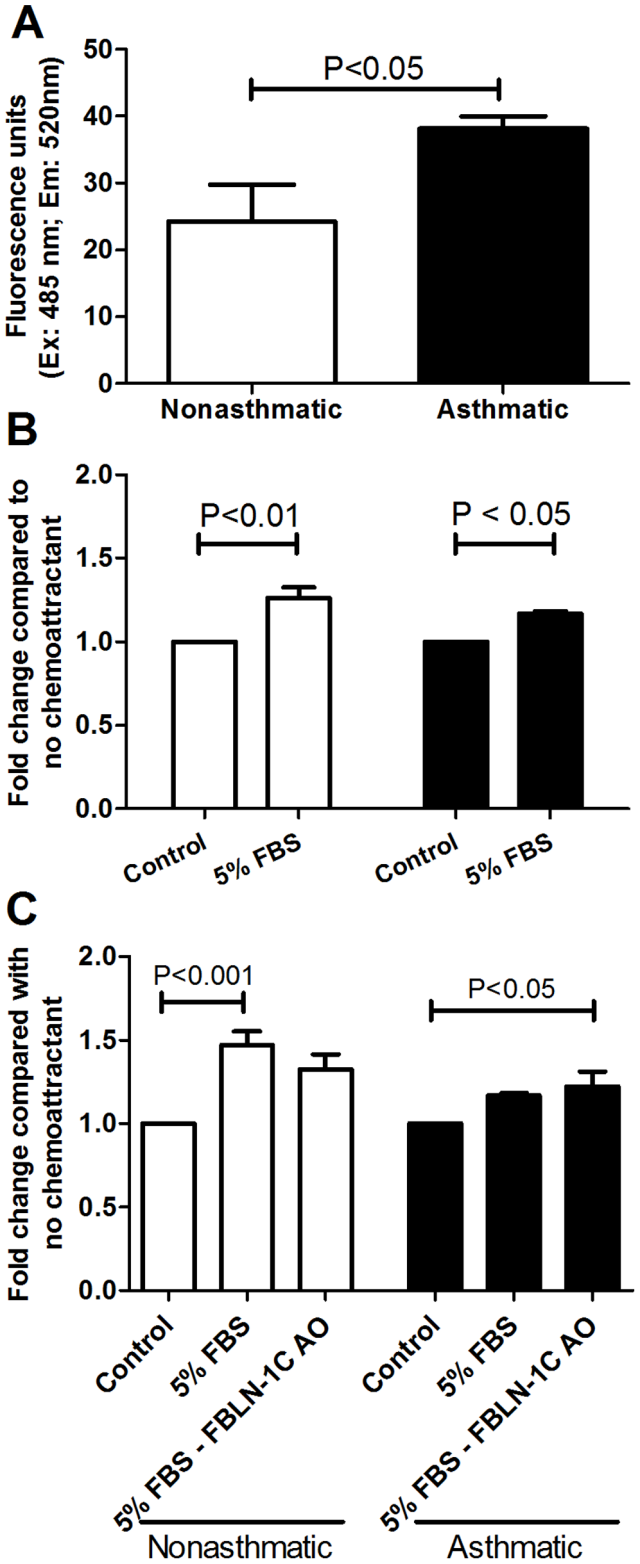
FBLN-1C does not alter migration of ASM cells. Migratory rates of non-asthmatic (white, n = 4–5) and asthmatic (black, n = 4–6) ASM reseeded on an ECM deposited by an asthmatic ASM cell line in the absence (A) or presence (B) of a chemoattractant (5% FBS). (C) ASM cells from non-asthmatic (n = 4) or asthmatic (n = 4–5) volunteers were reseeded onto an asthmatic ECM in which FBLN-1C was inhibited by a FBLN-1C specific AO and the effect on cell migration in response to 5% FBS was determined. Data are expressed as mean ± SEM.

Basal cell migration was increased in the asthmatic ASM cells when seeded on an asthma derived ECM (P<0.05) (figure 11A). However in the presence of a chemoattractant (5% FBS) chemotaxis of both non-asthmatic and asthmatic ASM cells occurred (P<0.01; P<0.001 respectively, Figure 11B). When FBLN-1C was silenced by the AO in the asthma derived ECM, migration of either cell type was not altered (figure 11C).

### Fibulin-1 contributes to the development of airway hyperresponsiveness in a murine model

**Figure 12:**
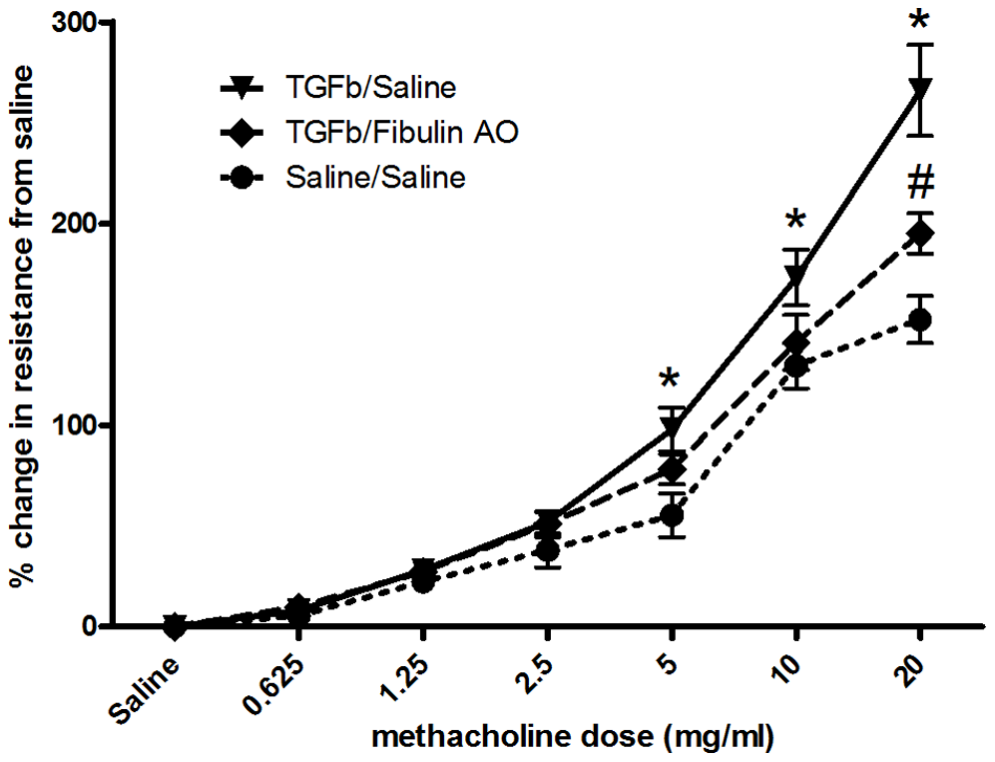
FBLN-1 contributes to the airway resistance of mice treated with TGFβ. Adult BALB/c mice (6–8 weeks old) were treated twice (day 0 & 1) with either recombinant human TGFβ or PBS. Seven mice were treated with the FBLN-1 AO daily for the first 7 days, and then 3 times weekly for the following three weeks. Specific airway resistance to methacholine was measured using whole body plethysmography. Data are expressed as percentage change compared with PBS and shown as mean ± SEM. n = 7 for all groups except saline/saline for which n = 6. TGFβ/Saline is significantly greater than saline/saline (denoted by *) at 5, 10 and 20 mg/ml methacholine, p<0.05, p<0.01, p<0.001 respectively. TGFβ/FBLN-1 AO is significantly reduced (denoted by #) compared with TGFβ/Saline at 20 mg/ml methacholine p<0.05.

In a murine model of airway hyperresponsiveness TGFβ increased the resistance (Figure 12) and decreased the compliance (data not shown) to methacholine in comparison with saline treated animals, demonstrating that AHR was induced (P<0.001, P<0.05 respectively). AHR induced by TGFβ was abolished (to similar levels to those seen in saline treated controls) when the mice were treated with the FBLN-1 AO. In the presence of TGFβ and the FBLN-1 AO resistance and compliance were not different from those in saline (Figure 12) or saline/FBLN-1 AO (data not shown) treated animals. Further, the scrambled AO did not alter airway resistance or compliance (P>0.05) in the presence or absence of TGFβ (data not shown).

## Discussion

In the present study, we demonstrated for the first time that the expression of FBLN-1 is increased in asthma. *In-vivo*, FBLN-1 levels were increased in BAL fluid and serum from asthmatic volunteers. *In-vitro*, TGFβ increased the expression of FBLN-1 in ASM cells. The increased FBLN-1 resulted in exaggerated proliferation and wound repair in asthma derived ASM cells which was reduced when only the FBLN-1C isoform was downregulated.

The current study demonstrates that FBLN-1 is differentially expressed in ASM from volunteers with and without asthma. Data obtained *ex-vivo* showed that FBLN-1 protein levels and FBLN-1C mRNA levels in ASM cells derived from asthmatics were higher than those derived from non-asthmatics. Our observation that TGFβ decreased FBLN-1D mRNA expression (figure 2D) was consistent with the findings of Laprise *et al.*, who reported reduced FBLN-1D mRNA expression in lysates from asthma derived bronchial biopsies compared with those derived from non-asthmatics [15]. The differential regulation of the specific isoforms of FBLN-1 indicates that they may have opposing or compensatory effects [33]. Given our findings of increased FBLN-1 protein in asthmatic ASM cells and the lack of information about FBLN-1C we chose to elucidate the role of FBLN-1C in this study.

We demonstrated that TGFβ not only increased FBLN-1 in asthma derived ASM cells, but also enhanced its deposition in the asthmatic ECM. The architecture of the asthmatic airway often undergoes prominent and permanent structural changes, including alterations of the molecular composition of the ECM. In particular, ECM protein deposition is increased in the *lamina reticularis* [34], resulting in basement membrane thickening [35]. Given that TGFβ levels may alter the ECM assembly process, it is likely that the raised TGFβ levels in the asthmatic airway may contribute to an increase in the ECM thereby augmenting the effects of airway remodeling.

Lung injury resulting from inflammation contributes to the remodeling process [36]. It is hypothesized that smooth muscle precursor cells from beyond the borders of the muscle or proliferating cells within the muscle bundles are recruited to the site of injury. In the present study, we examined the proliferative, migratory and wound closure properties of ASM cells derived from those with and without asthma. Notably, a recent *ex vivo and in-vitro* studies have indicated that proliferation is increased in asthma derived cells [3], [4], [22]. Our data support the current literature and, in addition, provide the first evidence that migration and wound closure are increased in asthmatic ASM cells. Wound healing involves synchronized events of cell proliferation, migration and differentiation [37]. Cell migration is a complex phenomenon and the wound assay has advantages over transwell or boyden chamber techniques which allow chemotaxis of cells freed from their native matrix to be investigated. In contrast, the wound assay allows for cell movement to be assessed in the absence of disruption of cell-cell or cell-ECM interactions and thus allows migration of a connected population of cells to be measured. If the increased cross-sectional area observed in airway remodeling in asthma is, at least in part, due to migration of the smooth muscle cells towards the lumen then it is likely that these cells are interacting with the surrounding environment and modulated by the ECM. In this study we showed cell proliferation and migration are abnormal in asthmatic ASM cells, suggesting that these events may contribute to aberrant repair processes in the asthmatic airway.

We confirmed that the ECM is another important factor in determining the properties of the ASM cells. Wound repair, cell proliferation and migration were all enhanced when either the ASM or the ECM was derived from asthmatic volunteers, a finding which is consistent with the hypothesis of previous studies [8], [34], [38]. We assessed the impact of FBLN-1 on cell proliferation, migration and wound repair.

Decreasing the level of FBLN-1C in the asthmatic ASM cell derived ECM components abrogated increased cell proliferation and wound repair, but not migration. We did not examine the effect of knocking out FBLN-1C in the non-asthmatic ECM as we were unable to induce FBLN-1 in this matrix with TGFβ. This indicates that the increased FBLN-1C levels in asthmatics contribute to wound repair and remodeling by increasing cell proliferation rather than cellular migration. We did not observe any additive effect on wound repair or proliferation when FBLN-1C in both ASM and ECM was downregulated (data not shown), which suggests that decreasing FBLN-1C in either component is adequate to inhibit these cellular processes.

Silencing FBLN-1C did not alter ASM cell migration in our study. This is in contrast with the findings of Qing *et al.*, who reported that a fibrosarcoma-derived cell line transfected with FBLN-1D had decreased invasive potential [39]. However, these authors studied FBLN-1D, whilst in our studies, we designed our AO to target FBLN-1C, which appears to have different properties to the other FBLN-1 isoforms [33]. In addition, a recent study showed FBLN-1C negatively regulated proliferation in human osteoblasts, indicating that the actions of the FBLN-1 isoforms are likely to be cell type specific [40]. Thus although FBLN-1 has been reported to decrease [41] and increase [32] wound repair in other organs, our study provides compelling evidence for a role for FBLN-1, specifically the 1C isoform, in increasing wound repair and remodeling in the airways.

AHR is a hallmark feature of asthma that has been linked to the pathological events associated with remodeling of the airway wall. TGFβ has also been linked to the mechanism underpinning AHR in asthma. In particular, TGFβ administration to the airways in mice induces peribronchial fibrosis that results in the development of AHR [42]. TGFβ induced AHR in the murine model we employed in our study. When these animals were treated with AOs targeting FBLN-1, TGFβ induced AHR was inhibited demonstrating a critical role for FBLN-1 in the mechanism of TGFβ induced AHR. Notably, this is, to our knowledge, the first report demonstrating that inhibition of a matrix protein reduces AHR, thereby providing direct evidence for the role of the ECM in regulating AHR in this model. This suggests that FBLN-1 may play a pivotal role in the processes that drive airway wall lesions in asthma that lead to enhanced airways reactivity. The regulated expression of FBLN-1 downstream of inflammation and its regulation of the remodeling/AHR axis identify the therapeutic potential of targeting this glycoprotein.

We were afforded the opportunity to study FBLN-1 levels in BAL fluid and serum from asthmatic volunteers both before and after administration of corticosteroids, and showed that treatment had no effect on FBLN-1. In our study population we did not observe a correlation between the level of FBLN-1 detected and the FEV_1_/FVC ratio. However, the range of asthma severity in our study may have been too narrow to enable the demonstration of such a relationship. If, as our results in the present study suggest, FBLN-1 is implicated in airway remodeling, then the lack of effect of corticosteroids on levels of FBLN-1 *in-vivo* highlights the need for treatments that target the structural changes which contribute to airway remodeling in asthma. The findings in our cell and animal based models indicate that FBLN-1 warrants further investigation as a novel target for therapeutic intervention to prevent airway remodeling.

## Supporting Information

Table S1 Patient demographics. Diagnosis, sample type, age, gender and predicted forced expiratory volume in one second (FEV_1_) of volunteers used in experiments.
(0.22 MB DOC)
