# Supplementary material for: Fibulin-1 Is Increased in Asthma – A Novel Mediator of Airway Remodeling?
Source: PLoS One. 2010 Oct 13;5(10):e13360. doi: 10.1371/journal.pone.0013360 (PMC2954173; doi:10.1371/journal.pone.0013360)
Supplement: Table S1 — Patient demographics. Diagnosis, sample type, age, gender and predicted forced expiratory volume in one second (FEV1) of volunteers used in experiments. (0.22 MB DOC) [file pone.0013360.s001.doc]

Table S1. Patient demographics. Diagnosis, sample type, age, gender and predicted forced expiratory volume in one second (FEV_1_) of volunteers used in experiments.

| **Patient number** | **Diagnosis** | **Sample type** | **Age** | **Sex** | **FEV_1_** | **Samples used in Experiments (Refer to footnote)** |
| --- | --- | --- | --- | --- | --- | --- |
| 1 | Asthma | Serum | 81 | Male | N/A | 4 |
| 2 | Asthma | Serum | 49 | Male | N/A | 4 |
| 3 | Asthma | Serum | 51 | Female | N/A | 4 |
| 4 | Asthma | Serum | 33 | Female | N/A | 4 |
| 5 | Healthy volunteer | Serum | 63 | Female | N/A | 4 |
| 6 | Asthma | Serum | 44 | Female | N/A | 4 |
| 7 | Asthma | Serum | 28 | Male | N/A | 4 |
| 8 | Asthma | Serum | 44 | Female | N/A | 4 |
| 9 | Asthma | Serum | 57 | Female | N/A | 4 |
| 10 | Asthma | Serum | 55 | Male | N/A | 4 |
| 11 | Asthma | Serum | 50 | Female | N/A | 4 |
| 12 | Asthma | Serum | 51 | Female | N/A | 4 |
| 13 | Asthma | Serum | 46 | Male | N/A | 4 |
| 14 | Healthy volunteer | Serum | 22 | Male | N/A | 4 |
| 15 | Healthy volunteer | Serum | 24 | Female | N/A | 4 |
| 16 | Healthy volunteer | Serum | 23 | Female | N/A | 4 |
| 17 | Healthy volunteer | Serum | 30 | Female | N/A | 4 |
| 18 | Asthma | Serum | 35 | Male | N/A | 4 |
| 19 | Asthma | Serum | 28 | Male | N/A | 4 |
| 20 | Asthma | Serum | 58 | Male | N/A | 4 |
| 21 | Healthy volunteer | Serum | 32 | Male | N/A | 4 |
| 22 | Asthma | Serum | 42 | Female | N/A | 4 |
| 23 | Asthma | Serum | 41 | Female | N/A | 4 |
| 24 | Healthy volunteer | Serum | 20 | Female | N/A | 4 |
| 25 | Asthma | Serum | 79 | Male | N/A | 4 |
| 26 | Asthma | Serum | 45 | Male | N/A | 4 |
| 27 | Asthma | ASM | 44 | Female | N/A | 4, 5 |
| 28 | Asthma | Serum | 46 | Male | N/A | 4 |
| 29 | Healthy volunteer | Serum | 61 | Female | N/A | 4 |
| 30 | Asthma | Serum | 42 | Male | N/A | 4 |
| 31 | Asthma | ASM | 25 | Male | N/A | 1, 3 |
| 32 | Asthma | ASM | 47 | Female | N/A | 1, 2, 4 |
| 33 | Asthma | ASM | 49 | Female | N/A | 5 |
| 34 | Healthy volunteer | ASM | 20 | Male | N/A | 1 |
| 35 | Healthy volunteer | ASM | 21 | Female | N/A | 1 |
| 36 | Healthy volunteer | ASM | 30 | Male | N/A | 1 |
| 37 | COPD | Serum | 82 | Male | N/A | 4 |
| 38 | Asthma | ASM | 43 | Female | N/A | 7 |
| 39 | Asthma | Serum | 40 | Male | N/A | 4 |
| 40 | Healthy volunteer | BAL fluid | 26 | Male | 92% | 4 |
| 41 | Asthma | ASM | 33 | Male | N/A | 1, 4, 7, 8 |
| 42 | Asthma | Serum | 43 | Female | N/A | 4 |
| 43 | Healthy volunteer | BAL fluid | 22 | Male | 97% | 4 |
| 44 | Asthma | ASM | 20 | Female | N/A | 2 |
| 45 | Healthy volunteer | BAL fluid | 25 | Female | 102% | 4 |
| 46 | Asthma | ASM, BAL fluid | 19 | Female | 97% | 1, 4 |
| 47 | Asthma | ASM, BAL fluid, serum | 18 | Female | N/A | 4 |
| 48 | Asthma | BAL fluid, serum | 20 | Female | 86% | 4 |
| 49 | Healthy volunteer | BAL fluid, serum | 33 | Male | 125% | 4 |
| 50 | Asthma | Serum | 19 | Male | - | 4 |
| 51 | Asthma | ASM, BAL fluid, serum | 26 | Male | 98% | 1, 2, 4 |
| 52 | Healthy volunteer | BAL fluid | 27 | Female | 100% | 4 |
| 53 | Asthma | ASM, serum | 20 | Male | N/A | 1, 4, 7, 8 |
| 54 | Asthma | ASM, BAL fluid, serum | 19 | Male | 79% | 1, 2, 4 |
| 55 | Asthma | ASM, BAL fluid, serum | 30 | Male | 75% | 1, 4 |
| 56 | Asthma | BAL fluid, serum | 20 | Male | 69% | 4 |
| 57 | Asthma | BAL fluid, serum | 19 | Female | 105% | 4 |
| 58 | Asthma | BAL fluid, serum | 25 | Male | 78% | 4 |
| 59 | Asthma | BAL fluid, serum | 21 | Male | 92% | 4 |
| 60 | Asthma | BAL fluid, serum | 29 | Female | 83% | 4 |
| 61 | Asthma | BAL fluid, serum | 20 | Male | 105% | 4 |
| 62 | Asthma | ASM, BAL fluid, serum | 25 | Male | 86% | 4 |
| 63 | Asthma | ASM, BAL fluid, serum | 25 | Male | 86% | 3, 4 |
| 64 | Asthma | BAL fluid, serum | 21 | Male | 62% | 4 |
| 65 | Asthma | ASM | 23 | Male | N/A | 5 |
| 66 | Asthma | ASM, BAL fluid, serum | 22 | Male | 100% | 4, 5 |
| 67 | Asthma | ASM, BAL fluid, serum | 27 | Male | 76% | 4, 5 |
| 68 | Asthma | BAL fluid, serum | 20 | Male | 80% | 4 |
| 69 | Asthma | BAL fluid, serum | 27 | Male | 76% | 4 |
| 70 | Asthma | ASM, BAL fluid, serum | 21 | Male | 98% | 3, 4, 5 |
| 71 | Healthy volunteer | BAL fluid, serum | 23 | Male | 80% | 4 |
| 72 | Healthy volunteer | BAL fluid | 19 | Male | 104% | 4 |
| 73 | Healthy volunteer | BAL fluid, serum | 20 | Male | 82% | 4 |
| 74 | Healthy volunteer | BAL fluid, serum | 31 | Male | 93% | 4 |
| 75 | Healthy volunteer | BAL fluid, serum | 22 | Female | 95% | 4 |
| 76 | Healthy volunteer | BAL fluid, serum | 22 | Male | 92% | 4 |
| 77 | Asthma | ASM | 56 | Female | 69% | 5 |
| 78 | Asthma | ASM | 60 | Male | 94% | 8 |
| 79 | Asthma | ASM | 38 | Male | 77% | 5 |
| 80 | Asthma | ASM | 40 | Male | N/A | 3, 5, 10 |
| 81 | Asthma | ASM | 27 | Male | N/A | 5, 10 |
| 82 | Carcinoma | ASM | 69 | Male | N/A | 5 |
| 83 | Healthy volunteer | ASM | 22 | Female | 91% | 1 |
| 84 | Asthma | ASM | 30 | Male | 100% | 1 |
| 85 | Asthma | ASM | 22 | Male | N/A | 1, 6 |
| 86 | Small cell carcinoma | ASM | 67 | Female | N/A | 1 |
| 87 | Primary pulmonary hypertension | ASM | 44 | Female | N/A | 1 |
| 88 | Large cell carcinoma | ASM | 54 | Female | N/A | 1 |
| 89 | Emphysema | ASM | 65 | Male | N/A | 1 |
| 90 | Non small cell carcinoma | ASM | 64 | Male | N/A | 1 |
| 91 | Non small cell carcinoma | ASM | 80 | Female | N/A | 1 |
| 92 | Emphysema | ASM | 48 | Female | N/A | 1 |
| 93 | Mass | ASM | 78 | Female | N/A | 2, 4 |
| 94 | Non small cell carcinoma | ASM | 63 | Male | N/A | 1 |
| 95 | complex congenital heart disease | ASM | 48 | Male | N/A | 2, 4 |
| 96 | Bronchiolitis obliterans | ASM | 45 | Male | N/A | 2, 4 |
| 97 | Emphysema | ASM | 64 | Female | N/A | 5 |
| 98 | Emphysema | ASM | 56 | Female | N/A | 5 |
| 99 | Mass | ASM | 66 | Female | N/A | 5 |
| 100 | Primary pulmonary hypertension | ASM | 50 | Male | 80% | 5 |
| 101 | Healthy volunteer | ASM | 29 | Male | N/A | 5 |
| 102 | Idoipathic Pulmonary Hypertension | ASM | 34 | Female | N/A | 5 |
| 103 | Non small cell carcinoma | ASM | 60 | Male | N/A | 5 |
| 104 | α1 antitrypsin deficiency | ASM | 56 | Female | N/A | 3, 5 |
| 105 | redo cystic fibrosis, multi resistant pseudomonas | ASM | 32 | Male | N/A | 5 |
| 106 | Pulmonary fibrosis | ASM | 55 | Female | 88% | 3 |
| 107 | Healthy volunteer | ASM | 20 | Female | N/A | 8 |
| 108 | Non small cell carcinoma | ASM | 46 | Male | 70% | 3 |
| 109 | Asthma | ASM | 23 | Female | 89% | 8, 10 |
| 110 | Carcinoma | ASM | 48 | Male | N/A | 3 |
| 111 | Healthy volunteer | ASM | 22 | Female | N/A | 7, 8, 10 |
| 112 | Carcinoma | ASM | 65 | Male | 72% | 8, 10 |
| 113 | Asthma | ASM | 21 | Male | N/A | 8, 9, 10 |
| 114 | Carcinoma | ASM | 50 | Female | N/A | 8, 9, 10 |
| 115 | Asthma | ASM | 31 | Female | N/A | 6, 7, 8, 9, 10 |
| 116 | Pulmonary hypertension | ASM | 15 | Male | N/A | 8, 10 |
| 117 | Non small cell carcinoma | ASM | 59 | Female | N/A | 7 |
| 118 | Chronic rejection | ASM | 22 | Male | N/A | 8, 9, 10 |
| 119 | Emphysema | ASM | 64 | Male | N/A | 7 |
| 120 | Transposition of the great arteries | ASM | 39 | Male | N/A | 7 |
| 121 | Asthma | ASM | 22 | Male | N/A | 7, 8 |
| 122 | Non small cell carcinoma | ASM | 66 | Male | N/A | 1 |
| 123 | Carcinoma | ASM | 64 | Male | N/A | 2, 4 |
| 124 | Bronchiectasis | ASM | 56 | Female | N/A | 6 |
| 125 | Healthy volunteer | ASM | 32 | Male | N/A | 6 |
| 126 | α1-anti-trypsin deficiency | ASM | 55 | Female | 28% | 6 |
| 127 | Pneumonitis | ASM | 59 | Male | 71% | 6 |
| 128 | Pulmonary fibrosis | ASM | 62 | Male | N/A | 6 |
| 129 | Asthma | ASM | 62 | Male | 85% | 6 |
